# Advanced monolayer and layer-by-layer nanocapsule systems for sustained release of carvacrol and *trans*-cinnamaldehyde against multidrug-resistant *Salmonella* in poultry

**DOI:** 10.1007/s00253-025-13573-4

**Published:** 2025-08-14

**Authors:** Samah Mechmechani, Kosar Zadeh, Neda Zadeh, Adem Gharsallaoui, Nahla O. Eltai, Tareq M. Osaili, Layal Karam

**Affiliations:** 1 Department of Nutrition Sciences, College of Health Sciences, QU Health, Qatar University, P.O. Box 2713, Doha, Qatar; 2 Univ Lyon, Université Claude Bernard Lyon 1, CNRS, LAGEPP UMR 5007, Villeurbanne, France; 3 Biomedical Research Center, QU health sector, Qatar University, P.O. Box 2713, Doha, Qatar; 4 Department of Clinical Nutrition and Dietetics, College of Health Sciences, The University of Sharjah, P.O. Box 27272, Sharjah, United Arab Emirates; 5 Research Institute for Medical and Health Sciences, University of Sharjah, P.O. Box 27272, Sharjah, United Arab Emirates; 6 Department of Nutrition and Food Technology, Faculty of Agriculture, Jordan University of Science and Technology, P.O. Box 3030, Irbid, 22110 Jordan

**Keywords:** Poultry, Nanoencapsulation, Monolayer, Layer-by-layer, Antimicrobial activity, Antimicrobial resistance

## Abstract

**Abstract:**

The rise of antibiotic-resistant *Salmonella* in poultry poses a significant public health challenge. This study assessed the efficacy of carvacrol and *trans*-cinnamaldehyde, in free and nanoencapsulated forms, as natural alternatives to chlorine for inactivating antibiotic-resistant *Salmonella* in chicken. While several studies have evaluated free *trans*-cinnamaldehyde and carvacrol, there is a notable lack of research on encapsulated forms, using different types of capsules for controlled release of these antimicrobials in food applications. Both compounds were encapsulated by spray-drying into monolayer nanocapsules using maltodextrin as the carrier material, and into layer-by-layer nanocapsules with an additional layer of low methoxyl pectin, to enhance their stability and prolong antimicrobial activity. Twelve treatment groups were evaluated, including controls (distilled water), chlorine (50 ppm), and various concentrations of carvacrol and *trans*-cinnamaldehyde in their free or nanoencapsulated forms. The combination of monolayer and layer-by-layer carvacrol nanocapsules at a total concentration of 4% resulted in the highest *Salmonella* reduction (3.7 log CFU/g) after 11 days, significantly outperforming all other treatments (*p* < 0.05). *Trans*-cinnamaldehyde-based treatments, whether free or encapsulated forms, demonstrated delayed but notable reductions (2.0–2.2 log CFU/g), followed by carvacrol treatments at 2% using layer-by-layer nanocapsules alone or in combination with monolayer nanocapsules (1.5 log CFU/g). Free or monolayer carvacrol at 2% achieved reductions of 1.1–1.3 log CFU/g, while chlorine (50 ppm) was the least effective, with a reduction of 0.9 log CFU/g. These findings underscore the potential of nanoencapsulated carvacrol and *trans*-cinnamaldehyde as sustainable, eco-friendly, and effective solutions for enhancing poultry safety, mitigating antimicrobial resistance, and meeting consumer preferences for natural food preservation.

**Key points:**

• *Carvacrol and trans-cinnamaldehyde treatments effectively reduced Salmonella in poultry*

• *Combination of monolayer and layer-by-layer carvacrol nanocapsules at 4% achieved the highest reduction (3.7 log CFU/g)*

• *Trans-cinnamaldehyde showed notable Salmonella reductions of 2.0–2.2 log CFU/g*

• *Chlorine was the least effective treatment, reducing Salmonella by 0.9 log CFU/g*

**Supplementary Information:**

The online version contains supplementary material available at 10.1007/s00253-025-13573-4.

## Introduction

The presence of *Salmonella* in poultry, particularly in chicken, is a major contributor to foodborne outbreaks and poses a significant public health challenge globally (Raut et al. 2023). Non-typhoidal *Salmonella* remains a leading cause of bacterial diarrhea worldwide, accounting for approximately 150 million infections and 60,000 deaths each year (CDC 2024). In the European Union, contaminated eggs and meat lead to over 91,000 *Salmonella* infections annually, resulting in economic losses of nearly 3 billion euros (EFSA 2025). Similarly, in the United States, *Salmonella* infections linked to poultry rank among the most costly foodborne diseases, with an estimated annual economic burden of approximately $2.8 billion (Scharff 2020).

Despite regional variations, the global burden of *Salmonella* is significant. The Middle East and North Africa rank third globally—behind African and South-East Asian regions—for *Salmonella enterica* in disability-adjusted life years (DALYs), with rates between 31 and 100 DALYs per 100,000 population (WHO 2024). Efforts to mitigate *Salmonella* are increasingly challenging with the alarming rise in antimicrobial resistance (AMR). This growing threat is driven by the widespread and inappropriate use of antibiotics in veterinary medicine, including cephalosporins, fluoroquinolones, and colistin (McEwen and Collignon 2018). Nearly half of foodborne *Salmonella* isolates now exhibit resistance to one or more antimicrobial agents, reducing the efficacy of first-line treatments like ampicillin and fluoroquinolones (Kiessling et al. 2002; Marchello et al. 2020; WHO 2018).

*Salmonella* contamination in poultry presents a complex challenge, occurring through multiple pathways during slaughter and carcass processing (USDA 2021). In many Asian countries, chlorine remains the most commonly used sanitizer for poultry meat (Yim 2025). While favored for its low cost, broad antimicrobial spectrum, and rapid effectiveness in reducing microbial loads on chicken, chlorine treatment reveals significant drawbacks (USDA  2021). The process can produce harmful disinfection byproducts (DBPs), which are potentially carcinogenic and toxic to humans and aquatic life (Diana et al. 2019; Fisher et al. 2014; USDA  2021). Moreover, emerging evidence suggests that some *Salmonella* strains are developing resistance to chlorine treatment, further compromising its long-term efficacy (Mohamed et al. 2015).

Faced with the limitations of traditional chlorine treatments, researchers are exploring plant-derived essential oils (EOs) as a promising alternative approach for food safety with minimal contribution to antimicrobial resistance (García-Salinas et al. 2018; Rao et al. 2019). Extracted from herbs like oregano, thyme, clove, and cinnamon, these natural antimicrobials present an effective solution for controlling foodborne pathogens (Milagres de Almeida et al. 2023). Cinnamaldehyde is classified as generally recognized as safe (GRAS), while carvacrol is an approved flavoring agent by the Food and Drug Administration (FDA USFaDA 2024). Bioactive compounds such as carvacrol and *trans*-cinnamaldehyde exhibit potential antibacterial properties (Milagres de Almeida et al. 2023). The potential of EOs extends far beyond antimicrobial action. In addition to enhancing food safety, extending shelf life and reducing lipid oxidation, they also exhibit promising anti-inflammatory and immune-stimulating properties (Grazul et al. 2023). However, the widespread application of EOs faces significant technical challenges. Their inherent hydrophobicity, high volatility, susceptibility to oxidation, low stability, and solubility limit their practical use in food applications (Sharma et al. 2022). Nanoencapsulation presents a promising solution to these challenges, by forming a protective barrier around bioactive compounds. This technique shields EOs from environmental degradation, reduces adverse effects on sensory attributes such as flavor and texture, enhances antimicrobial efficacy, and enables the controlled release of active compounds for prolonged efficacy (Ojeda-Piedra et al. 2022; Sharma et al. 2022). This approach not only extends antimicrobial action throughout a product’s storage period but also contributes to reducing food waste and enhancing food security (Ojeda-Piedra et al. 2022).

Several studies have investigated the use of free carvacrol and *trans-*cinnamaldehyde against microorganisms in chicken preservation (Karam et al. 2020; Karam et al. 2019; Osaili et al. 2021a; Porter et al. 2020; Shrestha et al. 2019; Wang et al. 2023). However, research on nanoencapsulated carvacrol and *trans-*cinnamaldehyde remains limited, with only a few limited studies on chicken (Shrestha et al. 2019), fish meat (Chuesiang et al. 2021), and selected food matrices (Sepúlveda et al. 2024). While free essential oils (EOs) have demonstrated strong antimicrobial properties, nanoencapsulation enhances their stability, enables controlled release, and prolongs antimicrobial activity. However, the application of nanocapsule technology, particularly multilayer capsules for controlled release, in poultry preservation, especially in combatting antibiotic-resistant *Salmonella*, remains largely unexplored. This gap highlights the critical need for further research in this area. Therefore, this study aimed to (i) evaluate the antimicrobial efficacy of free and encapsulated EOs (carvacrol and *trans*-cinnamaldehyde) against antibiotic-resistant *Salmonella* strains in poultry; (ii) compare the effectiveness of monolayer and layer-by-layer nanocapsules; and (iii) assess the effectiveness of essential oil-based treatments in comparison to traditional chemical chlorine treatments in poultry processing.

## Materials and methods

### Preparation of essential oils (EOs)

Carvacrol (C ≥ 98%, CAS number 499–75-2) and *trans-*cinnamaldehyde (TC 97%, CAS number 14371–10-9) were obtained from Sigma-Aldrich, France. Dimethyl sulfoxide (DMSO; 23,500.297, VWR, UK) was used to prepare the free carvacrol and *trans-*cinnamaldehyde emulsion at a final concentration of 2% (v/v). Chlorine (TS 5682) was obtained from Beyaz Kagit Supplier, Turkey.

### Formation and characterization of carvacrol and *trans*-cinnamaldehyde nanocapsules

The spray-drying method was utilized to produce two types of capsules containing carvacrol (C) and *trans-*cinnamaldehyde (TC), as outlined in our previous study (Yammine et al. 2023b). For the elaboration of monolayer (M) nanocapsules, sodium caseinate (SC) served as emulsifier, while maltodextrin (MD) acted as the carrier material. For layer-by-layer (L) nanocapsules, an additional layer of low methoxyl pectin (MP) was incorporated. Primary emulsions were prepared by fully hydrating SC in distilled water, pH adjusted to 3 and C and TC added to the stock solutions. The emulsions were homogenized at 15,000 rpm for 5 min and further processed through a microfluidizer (Microfluidics LM20, Microfluidics Corp., Newton, MA, USA) by passing the emulsion 3 times at 5 × 10^7^ Pa to produce more uniform distribution of small droplets. The synthesis of monolayer capsules involved adding MD stock solutions, resulting in final compositions of dry powders of 74.5% MD, 1.8% SC, and 18.6% C or TC. The main emulsions were mixed with MP and MD solutions for layer-by-layer capsules, yielding final compositions of dry powders of 72% MD, 1.8% SC, 1.8% MP, and 18.6% C or TC. The feed emulsions were injected into a laboratory spray-dryer (Mini Spray-Dryer Buchi B-290, Switzerland) under optimized conditions (Yammine et al. 2023b). The powder particles were stored in hermetically sealed containers until further analysis. The nanoparticle characterization was performed using a Zetasizer Nano ZS90 (Malvern Instruments, Malvern, UK) to measure the size, zeta potential (ζ potential) and polydispersity index (PDI) of encapsulated droplets. Prior to analysis, the nanoparticle powders were suspended in imidazole-acetate buffer (pH 3) and gently agitated. Mean diameters were expressed in nanometers (nm), and ζ potential values were expressed in millivolts (mV). These experiments were performed in triplicate.

### Growth conditions and cell suspension preparation

In this study, two antibiotic-resistant strains of *Salmonella* spp. isolated from retail chickens were used (Al-Hadidi et al. 2022). The antigenic formulae and serovars of the isolated strains were identified according to (ISO) (2014), which confirmed both as *S. enterica* serovar Infantis. The strains were stored in micro-vials preservative tubes (Microbank® microbial storage, Pro-Lab diagnosis, Richmond Hill, ON, Canada) at − 80 °C. For the experiment, bacterial suspensions of each strain were adjusted to a turbidity of 0.5 McFarland standard using DensiCHEK PLUS (BioMérieux, France) by inoculating a pure colony of *Salmonella* grown on a nutrient agar plate (HiMedia, USA) in phosphate buffer solution (PBS, Atom Scientific, England). Bacterial cocktail cultures were prepared by combining 1 ml of each bacterial strain suspension adjusted to 0.5 McFarland standard. The mixed culture was then decimally diluted with sterile buffered peptone water (BPW; Liofilchem, Via Scozia, Italy) to reach a final concentration of approximately 10^7^ CFU/ml.

### Antibiotic sensitivity evaluation

The sensitivity of the two *Salmonella* isolates to a relevant panel of antibiotics was evaluated. The bacterial suspension of each strain of 0.5 McFarland was swabbed onto a Mueller Hinton agar plate (MHA, HiMedia, USA) and allowed to dry completely. The agar surface was subsequently covered with antibiotic-impregnated discs (Liofilchem®, Roseto degli Abruzzi, Italy), with a maximum of six discs per plate, and the plates were incubated for 24 h at 37 °C (Al-Hadidi et al. 2022). The antibiotic susceptibility panel included fourteen discs (Table 1). To avoid false-negative results regarding colistin resistance, colistin susceptibility was assessed using the *E*-test (Liofilchem®, Roseto degli Abruzzi, Italy) according to the manufacturer’s instructions. The inhibition zone was measured in millimeters (mm) and interpreted according to the Clinical and Laboratory Standards Institute (CLSI) guidelines ((ISO) 2014).

**Table 1:** List of antibiotic discs used for the antibiotic susceptibility test

Antibiotic name	Antibiotic abbreviation	Concentration used (μg)
Tetracycline	TET	30
Ampicillin	AMP	10
Amoxicillin/clavulanic acid	AMC	20/10
Piperacillin/tazobactam	TZP	100/10
Ciprofloxacin	CIP	5
Trimethoprim/sulfamethoxazole	SXT	1.25/23.75
Cephalothin	KF	30
Ceftriaxone	CRO	30
Cefepime	FEP	30
Fosfomycin	FOS	200
Nitrofurantoin	F	300
Ertapenem	ETP	10
Meropenem	MRP	10
Chloramphenicol	C	30

### Preparation and treatment of chicken breast samples

Skinless chicken breast samples were purchased from retail market (Doha, Qatar) and were tested for *Salmonella* contamination according to the standard procedures outlined in ISO 6579 (ISO 2017). Only chicken samples that were free from *Salmonella* were used in this study. The chicken was carefully sliced into smaller, uniform pieces of 10 g each using a sterile, single-use scalpel, and cutting board. All procedures were carried out under aseptic conditions to prevent contamination. Then, 100 μl of the prepared bacterial cell mixture was spread onto the surface of 10-g chicken samples, resulting in an initial population of approximately 5 log CFU/g. The samples were left for 30 min to facilitate bacterial attachment before applying various treatments. Each piece of the chicken was treated with 1 ml of the appropriate antimicrobial treatment solutions spread on the top to cover all the surface of the chicken piece. Twelve treatments of the inoculated chicken were used in this study: CL: chlorine (50 ppm) which reflects industrial applications and meets regulatory recommendations (Fabrizio et al. 2002; Park et al. 2002; USDA 2024), FC2: free carvacrol at 2% (w/v), MC2: monolayer carvacrol nanocapsules at 2% (w/v), LC2: layer-by-layer carvacrol nanocapsules at 2% (w/v), MC1LC1: monolayer carvacrol at 1% (w/v) combined with layer-by-layer carvacrol at 1% (w/v), MC2LC2: monolayer carvacrol at 1% (w/v) combined with layer-by-layer carvacrol at 1% (w/v), FTC2: free *trans*-cinnamaldehyde at 2% (w/v), MTC2: *trans*-cinnamaldehyde monolayer nanocapsules at 2% (w/v), LTC2: *trans*-cinnamaldehyde layer-by-layer nanocapsules at 2% (w/v), MTC1LTC1: *trans*-cinnamaldehyde monolayer nanocapsules at 1% (w/v) combined with *trans*-cinnamaldehyde layer-by-layer nanocapsules at 1% (w/v), MTC2LTC2: *trans*-cinnamaldehyde monolayer nanocapsules at 2% (w/v) combined with *trans*-cinnamaldehyde layer-by-layer nanocapsules at 2% (w/v). Samples were treated with the antimicrobial solution for 60 s at room temperature (Osaili et al. 2021a; Sharma et al. 2013). Two negative controls were employed: control 1 consisted of samples treated with DMSO at a final concentration of 2% (w/v), while control 2 consisted of samples treated with nanoparticles free of EO. These controls were designed to assess the impact of DMSO and free nanoparticles on *Salmonella* growth and population count in chickens. Both controls showed no antimicrobial activity when used alone without the essential oil compounds. Following treatment, the samples were placed in polypropylene bags and kept at 4 °C for 1, 4, 7, and 11 days.

### Microbial enumeration

Following storage, individual samples (10 g) were transferred aseptically into sterile stomacher bags, and 90 ml of buffered peptone water (BPW; Liofilchem, Via Scozia, Italy) were added. Samples were then homogenized using a Stomacher (Stomacher® 400 Circulator, Seward, England) for 2 min. After homogenization, 0.1 ml of the appropriate decimal dilutions was plated in duplicate onto Hektoen agar (Liofilchem®, Roseto degli Abruzzi, Italy) for *Salmonella* counting, and the plates were incubated at 37 °C for 24 h. Plates of between 25 and 250 colony-forming units (CFU) were counted using a colony counter (Stuart Scientific, UK), and the data obtained were converted to log CFU/g.

### Statistical analysis

All treatments were conducted in duplicates and two replicates for microbiological testing. The statistical significance of the results was assessed using GraphPad Prism 10.0 software. For the antimicrobial treatment of chicken samples over an 11-day experiment, one-way ANOVA followed by Tukey’s multiple comparison analysis was used to evaluate differences in the means of log CFU/g between each treatment group on the same day and to compare means across different treatments for each day. Statistical significance was defined as *p* < 0.05.

## Results

### Carvacrol and *trans*-cinnamaldehyde nanocapsules characterization

The average ζ-potential, size, and PDI of the encapsulated droplets are summarized in Table 2. The results indicated that the zeta potential measurements of the dispersed droplets yielded average positive values of + 20.64 ± 4.02 and + 18.74 ± 4.87 mV for MC and MTC, respectively. In contrast, the average zeta potential for LC and LTC were negative, at − 5.46 ± 2.37 mV and − 4.95 ± 1.89 mV, respectively. Additionally, the sizes of LC (267.15 ± 4.23 nm) and LTC (289.24 ± 7.91 nm) were larger than those of MC (176.03 ± 3.65 nm) and MTC (169.11 ± 5.74 nm), with PDI values for all types of capsules remaining below 0.3.

**Table 2:** Average zeta potential (ζ-potential), size, and polydispersity index (PDI) of the two types of nanocapsules: monolayer (M) and layer-by-layer (L) for carvacrol (C) and *trans*-cinnamaldehyde (TC)

Type of capsule	ζ-Potential (mV)	Size (nm)	PDI
MC	+ 20.64 ± 4.02	176.03 ± 3.65	0.29 ± 0.03
LC	− 5.46 ± 2.37	267.15 ± 4.23	0.25 ± 0.02
MTC	+ 18.74 ± 4.87	169.11 ± 5.74	0.27 ± 0.02
LTC	− 4.95 ± 1.89	289.24 ± 7.91	0.19 ± 0.01

*MC*, monolayer carvacrol nanoparticles; *LC*, layer-by-layer carvacrol nanoparticles; *MTC*, monolayer *trans*-cinnamaldehyde nanoparticles; *LTC*, layer-by-layer *trans*-cinnamaldehyde nanoparticles

### Antibiotic resistance profile

Both bacterial strains exhibited resistance to tetracycline and cephalothin. *Salmonella* Infantis 1 also showed resistance to ampicillin and ciprofloxacin, with intermediate resistance to nitrofurantoin and chloramphenicol. In contrast, *S.* Infantis 2 was resistant to nitrofurantoin, but demonstrated intermediate resistance to ciprofloxacin and was sensitive to ampicillin and chloramphenicol. Additionally, both strains exhibited sensitivity to other antibiotics, including amoxicillin/clavulanic acid, trimethoprim/sulfamethoxazole, ceftriaxone, cefepime, fosfomycin, ertapenem, meropenem, and piperacillin/tazobactam (Table S1).

### Antimicrobial treatments

**Fig. 1:**
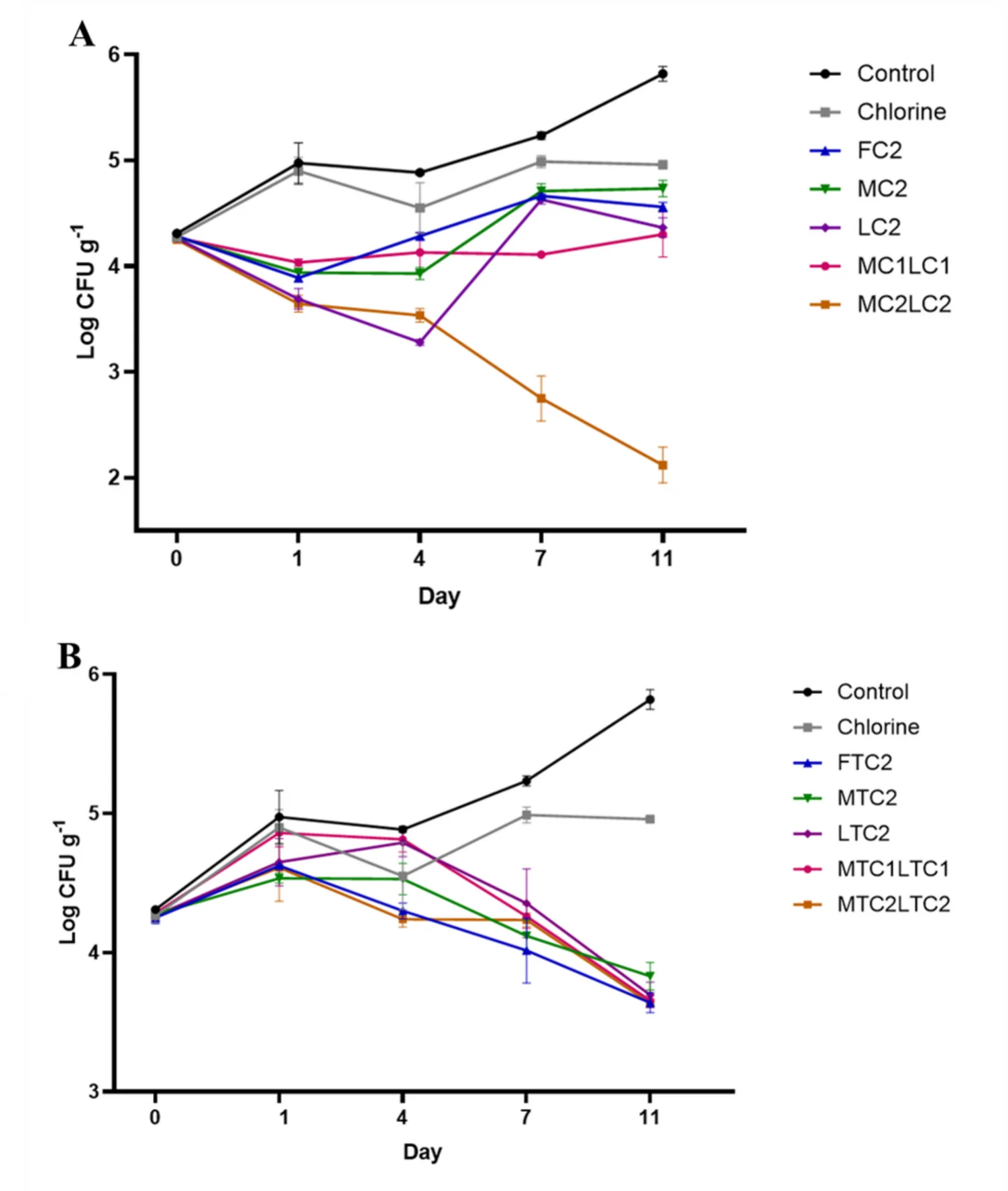
Effects of chlorine (50 ppm), carvacrol (**A**), and *trans-*cinnamaldehyde (**B**)-based treatments in free and nanoencapsulated forms on *Salmonella* counts (log CFU/g ± standard deviation) in chicken breast samples stored at 4 °C for 1, 4, 7, and 11 days. Sample treated with distilled water was used as control. FC2, free carvacrol at 2% concentration; MC2, monolayer nanoencapsulated carvacrol at 2% concentration; LC2, layer-by-layer nanoencapsulated carvacrol at 2% concentration; MC1LC1, combination of 1% monolayer and 1% layer-by-layer nanoencapsulated carvacrol; MC2LC2, combination of 2% monolayer and 2% layer-by-layer nanoencapsulated carvacrol; FTC2, free *trans-*cinnamaldehyde at 2% concentration; MTC2, monolayer nanoencapsulated *trans-*cinnamaldehyde at 2% concentration; LTC2, layer-by-layer nanoencapsulated *trans-*cinnamaldehyde at 2% concentration; MTC1LTC1, combination of 1% monolayer and 1% layer-by-layer nanoencapsulated *trans-*cinnamaldehyde; MTC2LTC2, combination of 2% monolayer and 2% layer-by-layer nanoencapsulated *trans*-cinnamaldehyde

The antimicrobial efficacy of chlorine and various carvacrol (FC2, MC2, LC2, MC1LC1, and MC2LC2) and *Trans-*cinnamaldehyde (FTC2, MTC2, LTC2, MTC1LTC1, and MTC2LTC2) treatments was evaluated over an 11-day period (Table S2), with initial microbial load of 4.3 log CFU/g across all samples and control (Fig. 1, Table S2).

The control samples treated with sterile water without any antimicrobials showed an increase in microbial populations from 4.3 log CFU/g at day 0 to 5.8 log CFU/g by day 11 during storage at 4 °C (Fig. 1, Table S2). A general trend of increase of *Salmonella* populations during storage occurred with a significant increase of 1.5 log CFU/g at day 11 compared to day 0 (*p* < 0.05).

The chlorine treatment exhibited limited antimicrobial efficacy, resulting in a slight but significant increase of around 0.7 log CFU/g in *Salmonella* populations at the last storage day (compared to day 0) (*p* < 0.05). In addition, the chlorine treatment significantly decreased *Salmonella* counts by 0.9 log CFU/g compared to the control at day 11 of storage (*p* < 0.05) (Fig. 1, Table S2).

Carvacrol-based treatments showed variable efficacy depending on the formulation days. FC2 was the least effective treatment, with a significant reduction only on day 1, followed by either stable or slightly increased counts during later storage (around 0.4 log on days 7 and 11) (Fig. 1A). MC2 showed strong initial reductions on day 1 and maintained similar *Salmonella* levels on day 4. However, its efficacy declined over time, with a slight but significant increase of 0.46 log CFU/g by day 11 (*p* < 0.05). In contrast, LC2 proved to be more effective, achieving significant reductions on both days 1 and 4, with sustained antimicrobial activity and no significant increase in *Salmonella* counts by day 11. The MC1LC1 treatment prevented any significant increase in *Salmonella* populations throughout the storage period. When comparing the four carvacrol treatments at 2% concentration to the control on day 4, the highest significant log reduction (*p* < 0.05) was observed for LC2 (1.6 log reduction), followed by MC2 (1 log reduction), MC1LC1 (0.8 log reduction), and the least effective treatment was FC2 (0.6 log reduction) (Fig. 1A). However, a different trend was observed with MC2LC2, where *Salmonella* populations consistently decreased during the storage period, reaching a significant reduction of 2.1 log CFU/g on day 11 compared to day 0 (*p* < 0.05) (Fig. 1A). By the last day of storage, MC2LC2 achieved a significant 3.7 log CFU/g reduction compared to the control (Fig. 1A) (*p* < 0.05).

*Trans-*Cinnamaldehyde treatments displayed delayed antimicrobial activity. In all *trans-*cinnamaldehyde-based treatments, whether free or encapsulated forms, *Salmonella* populations remained similar to their initial levels during the first days of storage (days 1 and 4). By day 7, the *Salmonella* population began to decrease, achieving significant reductions (around 0.6 log reductions) by day 11, compared to day 0 (*p* < 0.05) (Fig. 1B). By day 11 of storage, all *trans-*cinnamaldehyde-based treatments did not differ significantly, demonstrating a significant log reduction of 2.0 to 2.2, compared to the control (*p* < 0.05) (Fig. 1B).

Among all treatments used in this study, MC2LC2 treatment combining monolayer and layer-by-layer capsules at a total concentration of 4% was the most effective, achieving a reduction of 3.7 log CFU/g compared to the control at the last day of storage (*p* < 0.05). This was followed by *trans-*cinnamaldehyde treatments (2.0–2.2 log reduction) (*p* < 0.05), carvacrol treatments at 2% concentration using layer-by-layer capsules alone or in combination with monolayer capsules (1.5 log reduction) (*p* < 0.05), carvacrol treatments at 2% concentration using free or monolayer capsules (1.1–1.3 log reduction) (*p* < 0.05), and chlorine treatment (0.9 log reduction) (*p* < 0.05). However, no significant difference was noted among the treatments containing Carvacrol at a total concentration of 2% on the last day of storage.

## Discussion

### Nanocapsules characterization

The measurement of zeta potential for dispersed droplets revealed positive values for MC and MTC. This can be attributed to the positively charged surfactant used as an emulsifier at pH 3, which is below its isoelectric point (pHi ~ 4.5). In contrast, the zeta potential of LC and LTC shifted to negative values due to the presence of negatively charged carboxyl groups on the surface of the added polyanionic pectin chains (Kord Heydari et al. 2021). The low methoxylated pectin used in this study contains a higher concentration of negative charges, facilitating better binding and interaction with SC chains (Kord Heydari et al. 2021). The negatively charged pectin used in layer-by-layer nanoemulsions exerts a stronger electrostatic attraction on positively charged SC stabilized droplets, which explains why absolute zeta potential values for layer-by-layer emulsions were lower than those for monolayer emulsions. The reduced zeta potential values and increased interparticle attraction in layer-by-layer emulsions contribute to reduced particle stability. However, stability is not only influenced by zeta potential; factors such as emulsion viscosity, size, interfacial membrane properties, and density also play an important role (Yammine et al. 2023b). The size measurements revealed variations between monolayer layer-by-layer droplet emulsions, which could be due to the extra pectin layer in the LBL formulation. Notably, the PDI values of all droplets were below 0.3, suggesting a similar size distribution between formulations (Dantas et al. 2021). Nanoparticles with similar characteristics, including size and charge, containing thymol and carvacrol have been previously studied by Yammine et al. (2023a; 2022) and demonstrated significant antimicrobial activity against *S. enteritidis* and *L. monocytogenes* biofilms.

### Antibiotic resistance profile of *Salmonella* strains

Exploring the potential of EOs to control antibiotic-resistant *Salmonella* strains is vital for enhancing food safety. Carvacrol and cinnamaldehyde-based treatments were effective against antibiotic-resistant *Salmonella* strains. The two *Salmonella* strains used in this study are classified as multidrug-resistant (MDR) because they exhibited resistance to three different antibiotics belonging to different classes. Specifically, *Salmonella* 1 was resistant to tetracyclines (tetracycline), cephalosporins (cephalothin), beta-lactams (ampicillin), and fluoroquinolones (ciprofloxacin). Moreover, *Salmonella* 2 showed resistance to tetracyclines (tetracycline), cephalosporins (cephalothin), and nitrofurans (nitrofurantoin). Tetracycline is commonly used in animal feed for growth promotion and prophylaxis, indicating increased resistance rates in recent years (McEwen and Collignon 2018). Additionally, the overuse of other antibiotics, such as cephalosporins, nitrofurans, fluoroquinolones, and colistin in veterinary medicine further contributes to the rise of antimicrobial resistance (AMR) (McEwen and Collignon 2018; Ramos et al. 2017). Ampicillin and fluoroquinolones are among the first-line treatments for salmonellosis, making their overuse in livestock a significant factor in the increasing prevalence and dissemination of AMR (Marchello et al. 2020). The emergence and spread of antibiotic-resistant *Salmonella* are driven by factors such as misuse of antibiotics in healthcare and agriculture, poor hygiene practices, and the presence of mobile genetic elements like plasmids and transposons (Kabiru Olusegun and Samuel Oluwasegun 2017). Unregulated antibiotic sales and inappropriate prescription practices also contribute significantly to this issue (Kabiru Olusegun and Samuel Oluwasegun 2017). The mechanisms driving AMR in *Salmonella* include the transfer of resistance genes via plasmids, which can combine with virulence genes to produce highly resilient strains capable of thriving in antibiotic-rich environments (Su et al. 2004). Given the limitations of conventional treatments, natural antimicrobials, such as plant extracts, offer a promising alternative, as there have been no reported cases of resistance to EOs to date (Milagres de Almeida et al. 2023; Pisoschi et al. 2018; Rao et al. 2019; Trifan et al. 2020). However, Berdejo et al. (2020) showed that prolonged exposure to sublethal and lethal doses of carvacrol can further drive the emergence of resistant mutants of *Salmonella* Typhimurium. This highlights the need for further research on antimicrobial resistance in food pathogens and the importance of controlled use of food preservation strategies.

### Chlorine treatment

Chlorine remains a commonly used antimicrobial in the poultry industry due to its cost-effectiveness and ease of application, but its effectiveness can be limited in extended applications (Mohamed et al. 2015; USDA  2021). Chlorine treatment, applied at concentrations not exceeding 50 ppm, in accordance with the regulatory limits and safety considerations (USDA  2024), exhibited only modest reductions in *Salmonella* populations (0.9 log reduction as compared to the control by day 11) (*p* < 0.05). These results align with findings by Nagel et al. (2013), who reported reductions of less than 1 log CFU/ml of *Salmonella* populations on poultry carcasses using 40 ppm chlorine. Schambach et al. (2014) reported higher effectiveness with 50 ppm chlorine, achieving a reduction in *Salmonella* levels on chicken carcasses from 6 to 2.32 log CFU/ml after 45 min. Similarly, Aryal et al. (2024) reported reductions of ~ 4.86 log CFU/g in *Salmonella* enterica on spinach and ~ 3.97 log CFU/g on bell peppers, using a 100 ppm chlorine wash in 1 min. The significant differences in these results may stem from the type of food matrix being treated, as plant surfaces may interact differently with chlorine compared to poultry carcasses. Additionally, the concentration and contact time of chlorine play critical roles in its antimicrobial effectiveness. When chlorine is added to water, it generates free available chlorine in the form of hypochlorous acid and hypochlorite ions, with hypochlorous acid being the most effective at killing microorganisms (USDA  2021). Although chlorine is widely used in the poultry industry, its application comes with several limitations. In practice, chlorine can be corrosive to processing equipment at low pH levels, loses effectiveness at higher pH values, and its efficacy diminishes in the presence of organic matter and often requires longer contact times compared to alternative treatments (USDA  2021). Additionally, chlorine use can result in the formation of harmful disinfection byproducts (DBPs), such as haloacetic acids, sodium bromates, and trihalomethanes (Fisher et al. 2014; USDA  2021). These compounds are potentially carcinogenic and toxic to humans and aquatic life (Diana et al. 2019; Fisher et al. 2014). Exposure to DBPs has been associated with health risks like bladder cancer, respiratory issues, and reproductive problems, thereby raising significant environmental and public health concerns (Diana et al. 2019; Fisher et al. 2014; Pandian et al. 2022; Richardson and Postigo 2012; USDA  2021; Zheng et al. 2017). Moreover, some *Salmonella* strains have developed resistance to chlorine treatment, further questioning its long-term reliability as an antimicrobial solution (Mohamed et al. 2015). These limitations highlight the need for safer, more effective alternatives in food processing.

### Carvacrol-based treatments

Carvacrol has demonstrated significant antimicrobial effects in its free and encapsulated forms, with encapsulation technologies offering distinct advantages over free carvacrol in terms of stability, efficacy, and prolonged activity. Free carvacrol (FC2) at 2% effectively reduced *Salmonella* counts on day 1; however, this effect was not sustained, highlighting its limited long-term efficacy. In comparison, monolayer capsules of carvacrol (MC2) at 2% not only decreased *Salmonella* populations on day 1 but also maintained decreased population levels until day 4. This effect suggests a prolonged antimicrobial action due to the encapsulation process which protects carvacrol and enables its gradual release (Mechmechani et al. 2022a). Previous studies on free and encapsulated carvacrol revealed the potential for bacterial inhibition. Similar to our findings, Shrestha et al. (2019) showed that free and nanoemulsified carvacrol at 2% achieved equal reductions (around 4 log/sample) of *Campylobacter jejuni* on chicken skin. However, the free form achieved reductions immediately, while the nanoemulsified form achieved these reductions after 24 h. In another study, Engel et al. (2017) demonstrated that both free (0.331 mg/ml) and encapsulated (0.662 mg/ml) carvacrol used at minimum inhibitory concentration (MIC) completely inactivated *Salmonella* in biofilms within 10 min. Chen et al. (2021) highlighted the potential of using nanoencapsulated carvacrol to gradually reduce *E. coli O157:H7* on produce by 2.3 log CFU/cm^2^ over 14 days. Other studies also showed the potential of using free carvacrol and thymol for the preservation of chicken by achieving 2–3 log reduction of total aerobic counts at concentration of 0.4% and 0.8% (Karam et al. 2019). However, free carvacrol at the concentration of 0.1% achieved a reduction of < 0.5 log CFU/g in *Salmonella* on ground chicken after 12 days, suggesting the need for higher concentrations of carvacrol when applied on chicken (Porter et al. 2020). Overall, these studies indicate that carvacrol’s antimicrobial activity varies by concentration, form, culture, and food application, with both free and encapsulated forms showing antimicrobial performance in similar or different time frames. In addition, several studies have demonstrated the improved antibacterial activity of encapsulated EOs compared to their free forms against various bacterial strains (Khelissa et al. 2021; Mechmechani et al. 2022a, b); Yammine et al. 2022, 2023a). For instance, Kamimura et al. (2014) found that carvacrol encapsulated in hydroxypropyl-beta-cyclodextrin exhibited higher antimicrobial activity against foodborne pathogens *E. coli* and *S. enterica* than free carvacrol, indicating that encapsulation improves water solubility, thereby increasing contact between carvacrol and bacteria in the medium. Layer-by-layer carvacrol nanocapsules (LC2) at 2% demonstrated superior effectiveness, achieving initial reductions in *Salmonella* populations on day 1, and significant additional decrease by day 4. The extended antimicrobial effect observed with LC2 can be attributable to an additional pectin layer, which can slow the diffusion of carvacrol through the capsule shell (Das et al. 2022). This feature enables a more controlled release of the active compound over time, maintaining effective concentrations of carvacrol at the target site and ensuring a prolonged antibacterial action compared to other formulations (Das et al. 2022). The same 2 types of carvacrol nanocapsules were previously studied by Yammine et al. (2023b), who found that the monolayer nanocapsules released most of the active components within 2 h. Although the monolayer nanocapsules provide a quick release, they still offer controlled release properties, as their effects last longer than those of free carvacrol. In contrast, the layer-by-layer nanocapsules demonstrated a continuous and progressive release over 20 h. These findings confirm that monolayer and layer-by-layer nanocapsules provide quick and controlled release properties, respectively. However, the release kinetics reported by Yammine et al. (2023b) were evaluated in a medium that highlighted the hydrophilicity of the carrier polymers, resulting in rapid hydration of the capsules when suspended in PBS solution, which facilitated the swift diffusion of the encapsulated agents (Beirão-da-Costa et al. 2013; Li et al. 2022). In contrast, our study anticipated a slower release due to the use of a food matrix. By the end of the storage period on days 7 and 11, both FC2 and MC2 showed a significant increase in *Salmonella* populations, indicating a decline in their effectiveness over time. In contrast, LC2 maintained population levels on days 7 and 11 comparable to the initial counts, reflecting higher antimicrobial performance. The results highlighted the advantages of using LC2, as they provide more effective control over *Salmonella* growth throughout the storage period, compared to both FC2 and MC2. This sustained release capability is further elaborated by Yammine et al. (2022) who used the same types of monolayer and layer-by-layer carvacrol and thymol nanocapsules on *L. monocytogenes and S. Enteritidis* biofilms. They investigated the kinetics of release and delayed release of carvacrol from both types of nanocapsules. The findings indicated that monolayer capsules are more effective for initial surface disinfection due to the rapid release of EOs. In contrast, layer-by-layer nanocapsules played a crucial role in maintaining long-term disinfection efficacy through their sustained release mechanisms. Encapsulation is expected to protect active compounds from environmental factors such as light, humidity, oxygen, and pH, thus maintaining their bactericidal action for an extended period (Liolios et al. 2009; Reza Mozafari et al. 2008). Furthermore, the enhanced antimicrobial activity of encapsulated EOs is attributed to their reduced particle size and increased surface-to-volume ratio, which facilitate diffusion into microbial cells. Additionally, the encapsulation process improves the water solubility of EOs, enhancing their interactions with cell membranes—interactions that are challenging for free EOs (Cui et al. 2016; Granata et al. 2018; Moghimi et al. 2018). The combination of MC2 and LC2 exhibited distinct effects on *Salmonella* populations depending on the concentration used. At a lower concentration of 2% (MC1LC1), the population remained stable throughout the storage period, showing performance comparable to the LC2 treatment and resulting in a significant 1.5 log reduction compared to the control on the final day of storage (*p* < 0.05). However, at a higher concentration of 4% (MC2LC2), a consistent decline in *Salmonella* populations was observed throughout the storage period, achieving a significant reduction of 3.7 log CFU/g compared to the control by day 11 (*p* < 0.05). These findings underscore the enhanced preservation effect of this combination, especially at higher concentrations. The rapid release of carvacrol from MC2 can facilitate effective initial food preservation, while LC2 can provide long-lasting preservative effects through its sustained release properties. Similarly, Yammine et al. (2023b) explored the use of both monolayer and layer-by-layer nanocapsules of carvacrol and thymol for the treatment of *L. monocytogenes and S. enteritidis* biofilm, revealing that the combination of these 2 types of nanocapsules demonstrated the potential for effective surface disinfection. Monolayer nanocapsules provided a rapid release of EOs for immediate disinfection, effectively targeting and eliminating bacterial cells quickly. Meanwhile, layer-by-layer nanocapsules addressed any residual bacterial cells that may remain after the initial treatment. This dual approach ensured long-term disinfection and ongoing protection against microbial contamination on surfaces, achieving complete biofilm removal within 6 h of treatment. By strategically harnessing the strengths of both formulations, this approach results in comprehensive antimicrobial preservation of chicken.

### *Trans*-cinnamaldehyde-based treatments

The results of the TC treatments indicated a delayed antimicrobial effect. *Salmonella* populations remained generally stable on days 1 and 4 of storage, with significant reductions observed by day 11. All TC-based treatments demonstrated similar log reductions of 2.0 to 2.2 compared to the control (*p* < 0.05). These results demonstrated that both free and encapsulated forms of cinnamaldehyde exhibited comparable antimicrobial activity with a delayed reduction of bacterial growth. This suggests that encapsulation did not enhance the activity of cinnamaldehyde. This outcome may be attributed to the fact that the capsules released all their active components within 4 days. Given that the antimicrobial activity of cinnamaldehyde became apparent only by day 7, the minimal differences between the encapsulated and free forms may be related to the complete release of cinnamaldehyde before its bactericidal effect. Thus, the timing of release, the inherent properties and the mode of action of cinnamaldehyde are crucial in determining the overall antimicrobial efficacy observed in this study. Several studies reported that the impact of encapsulation on the antibacterial efficacy of lipophilic antimicrobial agents can vary, with some studies reporting an increase, similar or decrease in activity (Ayres Cacciatore et al. 2020; Buranasuksombat et al. 2011; Chang et al. 2012; Liang et al. 2012; Tian et al. 2016).

However, few studies have compared the antimicrobial activity of free and encapsulated forms of *trans-*cinnamaldehyde, and the available findings have been inconsistent. Similar to our research, applying *trans-*cinnamaldehyde nanoemulsions on eggs for 1 min achieved 2.0 to 2.5 log reductions of *Salmonella Enteritidis* (Allen et al. 2023). Chuesiang et al. (2021) reported similar efficacy for both nanoemulsion and bulk forms of cinnamon essential oil, with both achieving a gradual reduction of approximately 0.80 log CFU/g of *Salmonella* Typhimurium in Asian seabass fillet by day 8. However, Sepúlveda et al. (2024) found that encapsulation significantly increased the efficacy of cinnamaldehyde, resulting in reductions of 5.3, 3.3, and 1.8 log CFU/ml for *S. cerevisiae*, *L. innocua*, and *E. coli* in a protein-based apple juice beverage stored at 4 °C for 14 days. Additionally, Silva et al. (2018) observed that free cinnamaldehyde at 312 μg/ml (1MIC) and 624 μg/ml (2MIC) reduced the *Salmonella* Typhimurium biofilms from 8.03 log CFU/cm^2^ to 6.90 and 5.70 log CFU/cm^2^, respectively, in 1 h. However, it is essential to note that the performance of antimicrobials in culture may differ significantly from their effectiveness in food applications. This is due to complex interactions with food matrix components, making direct comparisons between the two contexts less accurate. Various factors can influence the antimicrobial activity of encapsulated components in nanoparticles, including interfacial properties, particle size, and the overall structure of the nanoparticles (Tian et al. 2016). Therefore, future research is needed to elucidate further how cinnamaldehyde in nanocapsules affects bacterial growth, which could provide valuable insights for optimizing its use as an antimicrobial agent.

### Carvacrol versus *trans*-cinnamaldehyde treatments

The differences in the antimicrobial effects of carvacrol and cinnamaldehyde are noteworthy. Carvacrol demonstrated an early antimicrobial effect, significantly reducing *Salmonella* populations by days 1 and 4. In contrast, the antimicrobial activity of cinnamaldehyde became evident only by days 7 and 11. This disparity can be attributed to the distinct modes of action of these two essential oils (Helander et al. 1998). Helander et al. (1998) investigated the mechanisms of action for both cinnamaldehyde and carvacrol, finding that while carvacrol disrupts the cell membrane of the Gram-negative bacterium *Salmonella* Typhimurium, cinnamaldehyde does not exhibit similar membrane-disrupting capabilities. This finding is supported by Di Pasqua et al. (2007), who also reported that cinnamaldehyde does not induce membrane collapse. Additionally, numerous studies have shown that carvacrol primarily targets the bacterial membrane. As a hydrophobic monoterpene, carvacrol readily penetrates bacterial cell membranes, disrupting their integrity and leading to the release of cellular contents, ultimately resulting in cell death (Cristani et al. 2007; Di Pasqua et al. 2007; Khan et al. 2017; Mechmechani et al. 2022a). This characteristic accounts for the rapid antimicrobial action observed with carvacrol. However, its volatility limits long-term efficacy as its active concentration decreases over time (Wang et al. 2023). However, research by Gill and Holley (Gill and Holley 2004, 2006a, b) suggested that cinnamaldehyde bactericidal action against *E. coli and L. monocytogenes* involves interaction with the cell membrane that rapidly inhibits energy metabolism. This disruption affects the proton motive force, causing leakage of small ions while larger components, such as ATP, remain intact, alongside inhibition of ATP generation and membrane-bound adenosine triphosphatase (ATPase) activity. Therefore, it is suggested that during the initial days of storage, *Salmonella* can utilize its stored energy reserves to maintain essential functions, allowing it to withstand the inhibitory effects of cinnamaldehyde. However, as storage continues, the sustained presence of cinnamaldehyde can increasingly impact the bacteria’s energy production. By day 4, as energy reserves diminish and the effects of cinnamaldehyde become more pronounced, *Salmonella* may reach a critical threshold where it can no longer sustain itself. Moreover, Pang et al. (2021) showed that the *trans*-cinnamaldehyde (TC) unique mode of action involves a stepwise disruption of bacterial membrane integrity through modulation of phospholipid biosynthesis, leading to progressive leakage of ions and metabolites. This gradual disruption can also allow TC to maintain antimicrobial activity over extended periods. This may explain why free TC demonstrated similar efficacy to encapsulated TC, as its inherent gradual mode of action allows it to sustain antimicrobial activity effectively even without encapsulation.

Studies comparing carvacrol and cinnamaldehyde have revealed variable antimicrobial activities across different food matrices. For instance, Wang et al. (2023) reported that free cinnamaldehyde achieved maximum *Salmonella* reduction on day 6, while free carvacrol achieved similar activity on day 2. Interestingly, Chen et al. (2015) reported that cinnamaldehyde exhibited better performance than carvacrol in peanut paste samples, achieving complete inactivation of *Salmonella* at 2500 ppm after 5 days, whereas carvacrol required higher concentrations (5000 ppm) with remaining viable cells (2.5 log CFU/g). On the other hand, carvacrol outperformed cinnamaldehyde when applied in chicken tawook, reducing *E. coli* O157:H7 by approximately 0.5 log CFU/g on day 7 at 1% concentration. In contrast, a higher concentration of cinnamaldehyde at 2% was needed to achieve the same reduction (Osaili et al. 2021a). Alternatively, Mattson et al. (2011) observed identical *Salmonella* log reductions in tomatoes using carvacrol and *trans*-cinnamaldehyde at 0.25–0.75% over an exact time frame. Additionally, Osaili et al. (2021b) reported equivalent antimicrobial effects of 2% carvacrol and 2% cinnamaldehyde on *Salmonella* in yoghurt-based marinades on camel meat (2.1–3.4 log CFU/g reduction) over the same time frames (days 4 and 7, respectively). This underscores the dynamic interplay between the type of antimicrobial agent, the form of the active component, the target bacterium, and the food matrix, and their evolving interactions over time.

## Conclusions

This study demonstrated the efficacy of natural compounds, specifically carvacrol and *trans*-cinnamaldehyde, as viable alternatives to chlorine for controlling antibiotic-resistant *Salmonella* in poultry. The combination of monolayer and layer-by-layer nanoencapsulated carvacrol at a concentration of 4% resulted in the most significant reduction in *Salmonella* counts, achieving a remarkable 3.7 log CFU/g reduction after 11 days of cold storage, compared to the control on the same day. These results underscore the potential of nanoencapsulation techniques to enhance food safety. Future research should focus on optimizing the formulation and application of these natural antimicrobials in various food processing contexts. Investigating the synergistic effects of combining essential oils with other preservation methods could further enhance their efficacy. Additionally, exploring the sensory characteristics, both before and after cooking, would provide valuable insights and strengthen the practical application of these treatments. Moreover, the use of Fourier transform infrared spectroscopy (FTIR) could provide insights into the interactions between these natural compounds and food matrices, helping to optimize their application in food safety. Ultimately, advancing these natural alternatives can contribute to mitigating antimicrobial resistance and improving food safety standards.

## Supplementary Information

Below is the link to the electronic supplementary material.

Supplementary file1 (DOCX 20.3 KB)

## Data Availability

No datasets were generated or analysed during the current study.

## Acronyms

AMR: Antimicrobial resistance
CFU: Colony-forming unit
DMSO: Dimethyl sulfoxide
EOs: Essential oils
FDA: Food and Drug Administration
GRAS: Generally recognized as safe
M: Monolayer
L: Layer-by-layer
C: Carvacrol
TC: *Trans*-Cinnamaldehyde
PDI: Polydispersity Index
SC: Sodium caseinate
WHO: World Health Organization
