# Supplementary material for: Advanced monolayer and layer-by-layer nanocapsule systems for sustained release of carvacrol and trans-cinnamaldehyde against multidrug-resistant Salmonella in poultry
Source: Appl Microbiol Biotechnol. 2025 Aug 14;109(1):182. doi: 10.1007/s00253-025-13573-4 (PMC12354115; doi:10.1007/s00253-025-13573-4)
Supplement: Supplementary file 1 — Supplementary file1 (DOCX 20.3 KB) [file 253_2025_13573_MOESM1_ESM.docx]

**Supplementary materials**

**Table S1.** Antibiotic resistance profile of *S.* Infantis 1 and *S.* Infantis 2

| **Family name** | **Antibiotic** | ***S.* Infantis 1** | | ***S.* Infantis 2** | |
| --- | --- | --- | --- | --- | --- |
|  |  | **IZ (mm)** | **Susceptibility** | **IZ (mm)** | **Susceptibility** |
| Tetracyclines | Tetracycline | Ø | R | Ø | R |
| Beta-lactams/penicillins | Ampicillin | 11 | R | 20 | S |
|  | Amoxicillin/clavulanic acid | 24 | S | 22 | S |
|  | Piperacillin/tazobactam | 24 | S | 25 | S |
| Fluoroquinolones | Ciprofloxacin | 20 | R | 27 | I |
| Trimethoprims | Trimethoprim/sulfamethoxazole | 16 | S | 20 | S |
| Cephalosporins (1st gen.) | Cephalothin | 9 | R | 10 | R |
| Cephalosporins (3rd gen.) | Ceftriaxone | 27 | S | 33 | S |
| Cephalosporins (4th gen.) | Cefepime | 32 | S | 30 | S |
| Phosphonic acid derivatives | Fosfomycin | 34 | S | 31 | S |
| Nitrofuran | Nitrofurantoin | 16 | I | 9 | R |
| Carbapenems | Ertapenem | 35 | S | 35 | S |
|  | Meropenem | 33 | S | 35 | S |
| Amphenicols | Chloramphenicol | 14 | I | 20 | S |
| Polymyxins | Colistin | 12 | S | 12 | S |

IZ: Inhibition Zone

Ø: No inhibition zone

R: Resistant, S: Sensitive, I: Intermediate

**Table S2:** Effects of Chlorine, Carvacrol, and Trans-Cinnamaldehyde based-treatments used in free or nano-encapsulated forms on the *Salmonella* counts (log CFU/g ± standard deviation) in chicken breast.

| **Treatment** | **Day 0** | **Day 1** | **Day 4** | **Day 7** | **Day 11** |
| --- | --- | --- | --- | --- | --- |
| **Control** | 4.31±0.02^aC^ | 4.98±0.17^aB^ | 4.89±0.06^aB^ | 5.24±0.04^aB^ | 5.82±0.07^aA^ |
| **Chlorine** | 4.27±0.05^aB^ | 4.90±0.11^abA^ | 4.55±0.21^abB^ | 4.99±0.05^abA^ | 4.96±0.04^bA^ |
| **FC2** | 4.28±0.04^aB^ | 3.89±0.01^dC^ | 4.28±0.04^bcB^ | 4.67±0.02^bcdA^ | 4.56±0.04^cdA^ |
| **MC2** | 4.27±0.04^aB^ | 3.94±0.00^dC^ | 3.93±0.05^cdC^ | 4.71±0.07^bcA^ | 4.73±0.07^bcA^ |
| **LC2** | 4.26±0.02^aA^ | 3.69±0.09^dB^ | 3.28±0.03^eC^ | 4.40±0.06^ceA^ | 4.37±0.09^cdA^ |
| **MC1LC1** | 4.27±0.01^aA^ | 4.03±0.04^cdA^ | 4.13±0.16^bcA^ | 4.11±0.00^eA^ | 4.30±0.18^dA^ |
| **MC2LC2** | 4.25±0.01^aA^ | 3.65±0.08^dB^ | 3.49±0.13^deB^ | 2.75±0.21^fC^ | 2.12±0.28^fD^ |
| **FTC2** | 4.25±0.04^aAB^ | 4.63±0.01^abA^ | 4.30±0.06^bcB^ | 4.01±0.23^eBC^ | 3.64±0.07^eC^ |
| **MTC2** | 4.27±0.04^aAB^ | 4.53±0.04^acA^ | 4.53±0.12^abA^ | 4.12±0.12^eBC^ | 3.83±0.10^eC^ |
| **LTC2** | 4.26±0.02^aB^ | 4.65±0.14^abAB^ | 4.79±0.10^aA^ | 4.24±0.08^deB^ | 3.69±0.09^eC^ |
| **MTC1LTC1** | 4.28±0.04^aA^ | 4.46±0.18^bcA^ | 4.47±0.16^abA^ | 4.26±0.08^deA^ | 3.65±0.05^eB^ |
| **MTC2LTC2** | 4.27±0.04^aA^ | 4.61±0.20^abA^ | 4.24±0.10^bcA^ | 4.23±0.08^deA^ | 3.64±0.01^eB^ |

Different superscript letters (a, b, c, d, e, f) indicate statistically significant differences (p < 0.05) between different treatment groups for the same day; different superscript letters (A, B, C, D) indicate statistically significant differences (p < 0.05) between different days for the same treatment group.

FC2, Free Carvacrol at 2% concentration; MC2, Monolayer Nano-encapsulated Carvacrol at 2% concentration; LC2, Layer-by-layer Nano-encapsulated Carvacrol at 2% concentration ; MC1LC1, Combination of 1% Monolayer and 1% Layer-by-layer Nano-encapsulated Carvacrol; MC2LC2, Combination of 2% Monolayer and 2% Layer-by-layer Nano-encapsulated Carvacrol; FTC2, Free Trans-Cinnamaldehyde at 2% concentration; MTC2, Monolayer Nano-encapsulated Trans-Cinnamaldehyde at 2% concentration; LTC2, Layer-by-layer Nano-encapsulated Trans-Cinnamaldehyde at 2% concentration; MTC1LTC1, Combination of 1% Monolayer and 1% Layer-by-layer Nano-encapsulated Trans-Cinnamaldehyde; MTC2LTC2, Combination of 2% Monolayer and 2% Layer-by-layer Nano-encapsulated Trans-Cinnamaldehyde.
